# Ethanol Lock Therapy (E-Lock) in the Prevention of Catheter-Related Bloodstream Infections (CR-BSI) after Major Heart Surgery (MHS): A Randomized Clinical Trial

**DOI:** 10.1371/journal.pone.0091838

**Published:** 2014-03-27

**Authors:** María Jesús Pérez-Granda, José María Barrio, Patricia Muñoz, Javier Hortal, Cristina Rincón, Pablo Martin Rabadán, Maria Sagrario Pernia, Emilio Bouza

**Affiliations:** 1 Department of Anesthesiology, School of Medicine, Universidad Complutense, Madrid, Spain; 2 Department of Clinical Microbiology and Infectious Diseases, School of Medicine, Universidad Complutense, Madrid, Spain; 3 Department of Pharmacy, Hospital General Universitario Gregorio Marañón, Madrid, Spain; 4 Medicine Department, School of Medicine, Universidad Complutense, Madrid, Spain; 5 Instituto de Investigación Biomédica Gregorio Marañón, Madrid, Spain; 6 CIBER Enfermedades Respiratorias-CIBERES (CB06/06/0058), Madrid, Spain; University of Pittsburgh Medical Center, United States of America

## Abstract

**Background:**

Lock-therapy with antimicrobials has been used for the treatment and prevention of catheter-related bloodstream infections (CR-BSI). Experiences with Ethanol-Locks (E-locks) have included therapeutic interventions with variable results. Patients undergoing Major Heart Surgery (MHS) are a high-risk population for CR-BSI.The aim of this study was to assess the efficacy and tolerance to E-Locks in the prevention of CR-BSI of patients undergoing MHS.

**Methods and Findings:**

This is an academic, prospective, randomized, non-blinded and controlled clinical trial assessing the incidence of CR-BSI of patients with E-locks (E-lock) and the tolerance to the procedure in comparison with patients receiving conventional catheter-care (CCC). Patients undergoing MHS with intravascular catheters for more than 48 hours were randomly assigned into treatment or control group by a computer-generated list of randomly assigned numbers. In the treatment group, all their catheter lumens were locked with an ethanol solution at 70% for two hours, every three days (E-Locks). The control group received conventional catheter-care (CCC).

Overall, 200 patients with 323 catheters were included in the study, which was stopped after 10 months due to adverse events. Of them, 179 catheters (113 patients) had E-Locks and 144 catheters (87 patients) were CCC. Euroscore Surgical Risk in both groups was 4.04 vs 4.07 p = 0.94 respectively. The results for the E-Locks and CCC were as follows: Incidence of CR-BSI/1000 days of exposure 2.1 vs 5.2 (p = 0.33), catheter tip colonization 14 (7.8%) vs 6 (4.2%) patients (p = 0.17), median length of hospital stay, 15 vs 16 days (p = 0.77). Seven patients (6.19%), all in the ethanol branch, had to discontinue the trial due to intolerance or adverse events.

**Conclusions:**

We do not recommend prophylaxis of CR-BSI with ethanol-lock on a routine basis in patients undergoing Major Heart Surgery.

**Trial Registration:**

Clinical Trials.gov NCT01229592

## Introduction

Patients undergoing Major Heart Surgery (MHS) are at a high risk of developing Catheter-Related Bloodstream Infections (CR-BSI) leading to a subsequent increase in morbidity and mortality [1]–[4].

Efforts developed to eliminate CR-BSI in patients hospitalized in Intensive Care Units (ICU's) have mainly concentrated on mixed ICUs and not specifically on patients following MHS, who usually require more catheters during more prolonged periods of time [5]–[7]. The programs addressed to eliminate CR-BSI episodes are mainly based on educational policies and on non-pharmacological measures and, although effective, these programs fail to lead to the disappearance of CR-BSI in most units and are often difficult to maintain for prolonged periods of time [8], [9].

The use of antibiotic lock solutions to treat CR-BSI has been relatively successful in specific situations, such as pediatric patients or home total parenteral nutrition related infections [10]–[12]. However, there is a concern about the potential risk of those solutions to induce the development of antibiotic therapeutic resistance [13]. Ethanol locks (E-locks) were greeted as a potential non-antibiotic alternative for the rescue of infected long-term catheters that were very difficult to substitute in different and disparate populations [14]–[18]. E-locks, however, have not been evaluated as a systematic procedure for the prevention of CR-BSI in the short-term use of catheters.

Our study is a prospective, randomized trial, evaluating the efficacy and tolerance to E-locks in the prevention of CR-BSI and catheter colonization in patients admitted to a specific MHS-ICU.

## Materials and Methods

Our institution is a general reference hospital with 1,550 beds and approximately 55,000 admissions/year during the study period. The Department of Cardiovascular Surgery is a large referral Unit that performs more than 500 MHS procedures annually. The start date of participant recruitment was February, 2,011.

### Study Design

Our study is a prospective, randomized, academic clinical trial, not funded by pharmaceutical or biotechnology companies. The patient' inclusion criteria were:

-Recent MHS admission with Central Vascular Catheters (CVC) inserted >48 hours.
-Age >18 years.
-No evidence or suspicion of CR-BSI at enrolment: No signs of infection neither general nor at catheter site entrance.
-No history of allergy or intolerance to ethanol or chronic liver disease.
-No pregnancy.

Patients who gave their informed consent were randomly assigned into two groups: Ethanol group (E-lock) and conventional catheter-care (CCC). Patients assigned to E-locks had CCC plus all catheter lumens locked with 1 ml of an ethanol solution at 70% for a period of 2 hours every three days, until catheter withdrawal. After the two hours, the ethanol was flushed through with 1 mL of saline solution.

Both groups received CCC according to standard recommendations and all catheters had split-septum connectors [19]. All catheters were withdrawn when clinically required and the catheter tips were systematically sent for culture.

### Preparation of the ethanol solution

The 70% ethanol solution was prepared by the Pharmacy Department as a sterile solution and was produced in 5 ml single dose vials after approval by the Spanish Agency for Medicines and Health Products and as established by the Spanish Legislation (Real Decreto 223/2004-Spain).

### Endpoints of the Study

The primary endpoint was the incidence of CR-BSI during the admission for Surgery.

### Secondary endpoints were

1.-The rate of colonization of the skin surrounding the catheter entrance, the hubs and the catheter tips in the E-lock and the CCC group.

2.-Antibiotic consumption in the two groups.

3.-Hospital stay, ICU stay and mortality in both groups of patients.

4.-Tolerance to the ethanol locks.

### Ethics

The protocol for this trial and the CONSORT checklist are available as supporting information, see Checklist S1 and Protocol S1. The Ethics Committee of our institution (Hospital General Universitario Gregorio Marañón) approved the study and all patients gave their written informed consent before inclusion in the study.

### Follow-up of patients

Patients were followed-up daily to check for the presence of infections and adverse reactions by both the physicians of the Department of Anaesthesia and by Infectious Disease specialists participating in the study. Clinical data were recorded according to a pre-established protocol and no further systematic surveillance cultures were performed.

### Pre-surgical information

Included epidemiological data, underlying diseases and standard scores (ASA, EuroSCORE, Charlson comorbidity index and APACHE II score on admission to the ICU).

### Surgical information

Included type of surgery, indication, duration, time of cardiopulmonary by-pass, aortic cross-clamp time and antimicrobial prophylaxis. Antimicrobial prophylaxis for surgery consisted of 2 g of cefazolin given before surgery and every 8 hours thereafter for a total of 3 doses (patients who were allergic to cefazolin received 1 g vancomycin before surgery).

### Postsurgical outcome

Enrolled patients were prospectively followed for the occurrence of CR-BSI until catheter withdrawal, hospital discharge or death. Outcome variables also included antimicrobial use measured as daily defined doses (DDD's), *Clostridium difficile* infection (CDI) episodes, length of ICU stay, ICU mortality, length of hospital stay and mortality at discharge.

For each intravascular line, the following data were recorded: type of catheter, insertion site, cause of removal, result of tip, skin and hub cultures and presence or absence of CR-BSI. Catheters were made of polyurethane and were non antibiotic coated.

### Data regarding adverse events

The following data were systematically collected in both populations after the ethanol or conventional locks were flushed: subjective tolerance, catheter obstruction, elevation of liver function enzymes and reasons for catheter withdrawal.

### Definitions

The definitions of CR-BSI are those detailed in the recent Clinical Practice Guidelines for the Diagnosis and Management of Intravascular Catheter-Related Infection [19]. For the purpose of this study we only accepted microbiologically proven CR-BSI considered when the same microorganism was recovered from blood and a catheter tip within less than 8 days. Catheter tip colonization, hub and skin colonization are defined as the presence of ≥15 colony forming units in the semiquantitative culture according to the roll plate technique recommendation [20].

### Statistical analysis

Considering that the previous incidence density of CR-BSI in our unit was 3.7 episodes/1000 catheter-days before the study, in order to be able to detect a difference of 25% between the two groups with 80% power and a 5% level of significance, we estimated that the sample size for the whole study should be 950 patients, divided equally in 475 in each arm.

Relationships between baseline variables were evaluated for the randomized groups. Basal comparisons between groups were established by clinical relevance according to the CONSORT recommendations [21]. The qualitative variables appear with their frequency distribution. The quantitative variables were reported as the mean and standard deviation (SD) and as the median and inter-quartile range (IQR) if their distribution was skewed. Continuous variables were compared using the Student's t test for normally distributed variables or median test for non-normally distributed variables. The Chi 2 or Fisher's exact test was used to compare categorical variables. The Kolmogorov-Smirnov test was used to check whether a variable had a normal distribution.

#### Multivariate analysis

CR-BSI incidence rates (event/1000 days of CVC) between E-lock and CCC group were compared by Cox regression including all the variables statistically or clinically associated with treatment in the univariate analysis. HR (hazard ratio) and 95% CI were also calculated. All statistical tests were two-tailed. The level of significance was set at p<0.05 for all the tests. The statistical analysis was performed with SPSS 12.0 and Stata 9.0 software.

## Results

**Figure 1:**
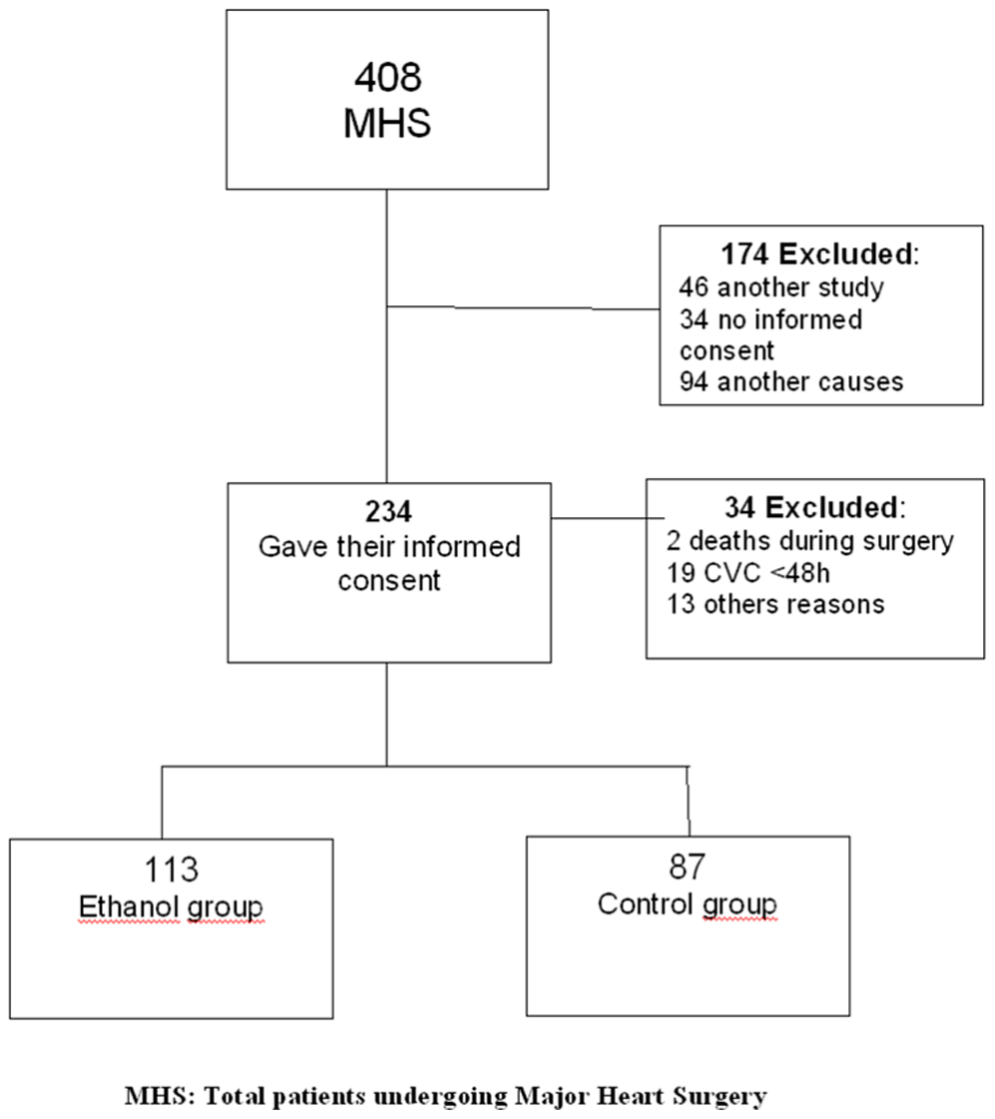
Selection of patients for the study.

The study was terminated early due to side effects. During the study period (February 28, 2011 to December, 31, 2011), 408 patients underwent MHS. Of them, 234 gave their informed consent to participate in the study. From these cases, 34 had to be excluded due to different reasons, including patients without CVC at the time of inclusion, death during surgery, early postoperative deaths, suspicion of infection immediately before the lock administration, or consent withdraw. The remaining 200 patients constitute the Intention to Treat Population (ITT) and 198 the Per-Protocol Population (PP). Only 2 patients failed to receive at least 1 dose of ethanol lock. The statistical analysis performed in both populations (ITT and PP) rendered similar results. The results of the ITT cohort are the ones shown in the manuscript. The patients in the study were randomized to the E-lock (113 patients) or CCC (87 patients) (Figure 1).

### Basal (Pre-Surgery) information in both populations

The characteristics of both populations and their co-morbidities are compared in Table 1. Sex, co-morbidities, underlying conditions, APACHE II score, ASA score and EuroSCORE (4.04±2.5 in the E-lock group vs 4.07±2.5 in the CCC group: p = 0.94) were similar in both groups of patients. The APACHE II score was respectively 8.89 (±2.7) for the E-lock and 8.86 (±2.5) for the Control Group, with no significant differences between them.

**Table 1:** Baseline characteristics and surgical variables of study patients in the overall population.

	Ethanol N = 113	Control N = 87	p value
**Preoperative**			
Mean age in years (SD)	67.3 (13.1)	65.2 (14.3)	0.29
Male sex (%)	62 (55)	47 (54)	0.90
Underlying conditions (%):			
Myocardial infarction	16 (14.2)	13 (14.9)	0.87
Congestive heart failure	15 (13.3)	7 (8.0)	0.24
CNS disorder	2 (1.8)	3 (3.4)	0.45
COPD	15 (13.3)	22 (25.3)	0.03
Renal dysfunction	7 (6.2)	9 (10.3)	0.28
Liver disease	4 (3.5)	6 (6.9)	0.28
Diabetes mellitus	30 (26.5)	29 (33.3)	0.29
Peptic ulcer disease	2 (1.8)	3 (3.4)	0.46
Malignant neoplasm	12 (10.6)	4 (4.6)	0.12
Non-fatal underlying disease (%)(McCabe criteria) [40]	111 (98.2)	85 (97.7)	0.79
Mean comorbidity index (±SD) Charlson's criteria) [41]	1.29 (1.64)	1.38 (1.32)	0.51
Mean Apache II score (±SD)	8.88 (2.7)	8.86 (2.59)	0.97
Patients with ASA Score >3 (%)	112 (99.1)	84 (96.6)	0.19
Mean EUROSCORE[42] (±SD)	4.04 (2.5)	4.07 (2.5)	0.94
**Operative data**			
Valve replacement (%)	58 (51.3)	53 (60.9)	0.17
Coronary artery bypass grafting (CABG) (%)	30 (26.5)	16 (18.4)	0.17
Mixed (valve and CABG) (%)	20 (17.7)	11 (12.6)	0.32
Mean CPBT (min) (SD)	122.9(54.0)	117.1(63.6)	0.49
Mean aortic cross-clamp time (min) (SD)	80.3 (35.4)	75.1 (45.1)	0.36
Mean total surgery time (min) (SD)	253.8 (78.6)	256.7 (88.5)	0.80

Abbreviations: COPD: chronic obstructive pulmonary disease; CNS: Central Nervous System; CABG: coronary artery by-pass grafting; CPBT: cardiopulmonary by-pass time.

### Data of the surgical procedure

Type of surgery, mean time on cardiopulmonary by-pass, aortic cross-clamp time and antimicrobial prophylaxis were similar in both groups (Table 1).

### Vascular access

Both populations showed no significant differences in the total number of catheters, mean number of catheters per patient, location of catheters, days of exposure to catheters, use of lipid or parenteral nutrition and other parameters (Table 2).

**Table 2:** Characteristics of Vascular Access in both groups.

	Ethanol N = 113	Control N = 87	p value
Total catheters (n)	179	144	0.44
Cultures taken (n)	1289	1147	0.37
Patients with Positive cultures	31	26	0.70
Total Catheter exposure (days)	955	805	—
Days of catheter exposure. Median (IQR)	6 (4–8)	7 (5–9)	0.55
Type of catheter (%):			0.58
Conventional	122 (68.2%)	94 (65.3)	
Swan-Ganz	57 (31.8)	50 (34.7)	
Location (%):			0.34
Jugular	172 (96.1)	141 (97.9)	
Subclavian	7 (3.4)	3 (2.1)	
Total Parenteral Nutrition (%)	6 (3.4)	6 (4.2)	0.70
Reasons for catheter withdrawal (%):			0.85
End of use	166 (92.8)	135 (93.8)	
Obstruction	5 (2.8)	4 (2.8)	
Suspicion of infection	3 (1.7)	3 (2.1)	
Adverse events	2 (1.1)	0	

### Lock procedures

The lumens of catheters in the ethanol group were locked sequentially every 3 days with 1 ml of 70% ethanol solution. After the 2 hour locks, the lumens were flushed with 5 ml of normal saline.

The total number of locks (all catheters lumens) that were made with ethanol was 338, with a range that varied between 1 and 25 locks per patient (median 2.0). Dwell time was 2 hours, uniformly in all cases.

### Primary endpoints

Post-surgical outcomes of both populations are summarized in Table 3. The number of episodes of CR-BSI was 2 episodes in the ethanol group (2.1 episodes/1,000 days of exposure) and 4 episodes in the control group (5.0 episodes/1000 days of exposure). The calculated incidence rate ratio was 0.42 (95% confidence interval 0.04–2.90), which implies a non-significant reduction of 58% for patients treated with ethanol locks (p = 0.33). The episodes of CR-BSI occurred in days 4 to 44 postoperatively and the etiology of the episodes is shown in Table 3.

**Table 3:** Clinical outcome in all randomized patients

	Ethanol N = 113	Control N = 87	p value
**Catheter colonization:**			
Overall (%)	39 (21.8%)	36 (25%)	0.32
Skin (%)	30 (16.8)	32 (22.2)	0.21
Skin colonization (density/1000 catheter-days)	39.52	49.53	0.38
Hub (%)	4 (2.2)	3 (2.1)	0.92
Hub colonization (density/1000 catheter-days)	4.40	3.82	0.87
Tip colonization (%)	14 (7.8)	6 (4.2)	0.17
Tip colonization (density/1000 catheter-days)	15.26	8.0	0.19
**CR-BSI:**			
Episodes (%)	2 (1.8)	4 (4.6)	0.24
CR-BSI Density per 1000 catheter-days	2.17	5.24	0.33
**Other infections (%):**			
Urinary tract infection	7 (6.2)	7 (8.0)	0.61
Bacteremia	6 (5.5)	6 (7.1)	0.64
Ventilator associated pneumonia	2 (1.8)	3 (3.4)	0.45
Surgical Wound Infection	3 (2.7)	0 (0)	0.12
*Clostridium difficile*-infection	2 (1.8)	1 (1.1)	0.63
**Days of Stay Median (IQR):**			
ICU	5 (3–7)	6 (3–7)	0.75
Hospital	15 (12–25)	16 (13–28)	0.77
Mortality in hospital (%)	7 (6.2)	7 (8.0)	0.61
Overall DDD's of AA during hospital stay. Mean (SD)	7.99 (18.6)	9.11(16.5)	0.65

Abbreviations: IQR: interquartile range; DDDs: daily defined doses; AA: Antimicrobial Agents.

### Secondary endpoints

We were not able to demonstrate differences in the secondary endpoints of the study (Table 3), including days in the ICU and hospital stay, days of catheter exposure and number of colonized catheters. No differences were detected between both groups in the incidence of other infections, antibiotics consumption, incidence of CDI, ICU or hospital mortality (Table 3).

### Adverse events

The adverse events of both groups are summarized in Table 4. Obstruction of catheters that required catheter withdrawal occurred in 5 patients in the ethanol group and in 4 patients in the control group (p = 0.87) (Table 4). In 7 patients the ethanol locks could not be completed, in five of them due to hemodynamic instability (high doses of inotropics). One catheter in the ethanol group had to be removed because of the rupture of 1 of the 3 lumens (Table 4).

**Table 4:** Adverse events.

	Ethanol N = 113	Control N = 87	p value
Discontinuation of the study compound	7	0	0.018
Severe immediate adverse events	2	0	0.21
Rupture of the catheter lumen	1	0	0.37
Patients with ALAT–ASAT elevations (double than normal value)	42	33	0.91

In the five minutes following the flushing of the lock solutions, 2 patients in the ethanol group presented important adverse events. One had chest pain accompanied by high blood pressure, without any other hemodynamic or electrocardiographic changes. The chest pain disappeared rapidly after the catheter was withdrawn. The second patient developed intense headaches and photopsies. The flushing was stopped and the catheter withdrawn. The catheter tip from this patient was inappropriately placed in the upper jugular vein. The comparison of liver enzymes, ALAT and ASAT was not significantly different between both groups (Table 4).

### Microorganisms isolated from colonization and CR-BSI episodes

For the purpose of this study, all removed catheters were sent for culture to the Microbiology Department (314 of 323). Overall, 20 patients showed a positive semiquantitative count of the catheter tip. Of these, 14 belonged to the E-lock group, while 6 were in the CCC group (p = 0.17).

Surveillance cultures of the peri-catheter skin and hubs were systematically done until catheter removal. The number of patients with one or more positive surveillance cultures was not significantly different in the population treated with ethanol (31/113) than in the conventional treatment population (26/87) (p = 0.70).

The microorganisms present in significant counts in surveillance cultures are summarized in Table 5. No significant differences were found in the microorganisms causing CR-BRSI or colonization in both groups.

**Table 5:** Microorganisms present in significant counts in surveillance cultures and CR-BSI.

	Ethanol N = 113	Control N−87	p-value
CR-BSI (6):	2	4	0.82
Gram positive cocci (%)	0	1 (1.14)	
*Enterobacteriaceae* (%)	2 (1.76)	2 (2.29)	
GNNFR (%)		1 (1.14)	
Fungi (%)	0	0	
Colonized patients (57) (1species per patient):			0.83
Gram positive cocci (%)	35 (30.97)	27 (31.03)	
Enterobacteriaceae (%)	4 (3.53)	2 (2.29)	
GNNFR (%)	0	1 (1.14)	
Fungi (%)	1 (0.88)	1 (1.14)	

GNNFR: Gram negative non-fermenting rods.

### Multivariate analysis

In the multivariate analysis the difference of the incidence density of CR-BSI, between the E-lock and CCC groups was not significant. The number of patients needed to be treated with ethanol-locks to prevent one CR-BSI episode was 345.

## Discussion

Our study shows that despite a trend in the reduction of CR-BSI, the use of ethanol locks for the prevention of CR-BSI in patients undergoing MHS should not be recommended. It is cumbersome and associated with serious adverse events.

In recent years, different campaigns have emphasized the need to drastically reduce CR-BSI and have set zero tolerance concept to those infections [8], [22]. Achieving and maintaining the zero incidence, however, proved to be a very difficult or impossible task, even in situations and institutions that made very serious and obstinate efforts [23], [24]. The search for alternative, non-educational procedures to contribute to a zero incidence remains pertinent, particularly in populations with many catheters and at a high risk for CR-BSI, as it is the case of patients undergoing MHS [25].

The use of high concentrations of antibiotics in the catheter lumens (lock-therapy) proved to be effective both in the conservative treatment management of infected catheters [11], [26] and also as a prophylactic approach in certain situations [27], [28]. Antibiotics, however, have different activities against different microorganisms, their penetration in the catheter biofilm is highly variable and the long-term use of antibiotic-locks, mainly in prophylaxis, may lead to the development of antimicrobial resistance [13], [29].

Ethanol is easy to produce and has a broad spectrum of antimicrobial activity, including bacteria and *Candida* [28], [30]–[32]. Studies of ethanol lock as a therapeutic agent for the treatment of CR-BSI are frequently based on case reports or very short series [33] and usually selected for treatment of episodes caused by “easy to treat” microorganisms such as Coagulase Negative Staphylococcus. Under these circumstances, eradication of infections were obtained in up to 86% of the episodes in the McGrath's study [17]. The tolerance to alcohol in those studies was generally good [33], [34] but thrombosis and other adverse events were reported [15], [35].

Data regarding the efficacy of ethanol locks in the prevention of CR-BSI is scarce. A meta-analysis in pediatric patients with short bowel syndrome and long-term catheters carried out by Oliveira et al [16], found 4 retrospective studies in this population. In comparison with heparin-locks, ethanol locks reduced the CR-BSI-rate by 81% and the need of catheter replacements by 72%. Overall, 108 to 150 catheter days of ethanol exposure were necessary to prevent one CR-BSI episode and 122 to 689 days of exposure prevented one catheter replacement.

In the case of hematologic patients with tunneled catheters, a randomized, double-blind, placebo-controlled trial compared ethanol locks for 15 minutes per day with a placebo. No significant differences were found between both groups in the incidence of CR-BSI, but more patients receiving ethanol discontinued lock-therapy (11 of 226 versus 1 of 222; p = 0.006) or continued with decreased lock-frequency (10 of 226 versus 0 of 222; p = 0.002) due to non-severe adverse events. In patients allocated to ethanol locks, one device had to be removed because of the rupture of 1 of the 3 catheter lumens. Facial flushing occurred in 39 out of the 226 patients with ethanol, compared to 17 out of the 222 patients with placebo (p<0.001) and feelings of dizziness/drowsiness occurred in 41 and 10 patients with ethanol or placebo respectively (p<0.001) [36].

Occasional information indirectly suggest the effectiveness of ethanol in preventing infections in the subgroups of patients on Total Parenteral Nutrition [15], [34] and in other patients [37] but different adverse events including thrombosis and catheter dysfunction were also reported [15], [35], [38].

Our population, of patients undergoing Major Heart Surgery, is a uniform but different population, frequently hemodynamically unstable and with highly needed central lines. The number of CR-BSI was reduced by 58% in patients receiving ethanol in our study, but the differences did not reach statistical significance, probably due to the early stop of our study and the consequent low power. Our calculations show that to prevent an episode of CR-BSI in our patients, 345 cases would have to receive prophylactic ethanol.

We selected as a primary end-point CR-BSI instead of CLABSI (Central Line Associated Bloodstream Infection). CLABSI is an approximate concept useful for epidemiological surveillance, while CR-BSI requires the confirmation with catheter cultures of the etiology and origin of CR-BSI. In our study the culture of catheters was uniformly performed upon withdrawal and the risk to miss cases was totally avoided. Furthermore, we have recently demonstrated that the trends of CLABSI and CR-BSI in the very unit in which we carried out this study [39].

The principal limitation of this trial is that we only enrolled the population undergoing Major Heart Surgery and this data cannot necessarily be extrapolated to other populations. Also very importantly, due to the detection of adverse events in the ethanol branch, we had to stop the trial after 10 months and our study was underpowered to detect a significant reduction of the CR-BSI episodes.

The limited and doubtful reduction of CR-BSI with the use of ethanol locks, the adverse events, the workload of the procedure for the nursing staff, the need for a high frequency of catheter manipulation and the requirement to lock highly needed lines during 2 hours, were among the reasons to stop our study. We do not recommend further studies with ethanol lock in the prevention of CR-BSI in patients following MHS.

## Supporting Information

Checklist S1 **CONSORT Checklist.**
(DOC)

Protocol S1 **Trial Protocol.**
(DOC)

Protocol S2 **Spanish Translation of Protocol S1.**
(DOC)
