# Supplementary material for: Ethanol Lock Therapy (E-Lock) in the Prevention of Catheter-Related Bloodstream Infections (CR-BSI) after Major Heart Surgery (MHS): A Randomized Clinical Trial
Source: PLoS One. 2014 Mar 27;9(3):e91838. doi: 10.1371/journal.pone.0091838 (PMC3967996; doi:10.1371/journal.pone.0091838)
Supplement: Protocol S1 — Trial Protocol. (DOC) [file pone.0091838.s002.doc]

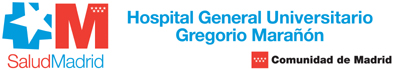


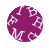


**“Study of *Ethanol Lock Therapy (E-Lock) in the prevention of Central Venous Catheters-Related Bloodstream Infection (CR-BSI)*”**

**Servicio de Microbiología Clínica y Enfermedades Infecciosas**

**Care Unit after Major Heart Surgery**

**Hospital General Universitario Gregorio Marañón**

**Nº DE PROTOCOL: Nº DE PACIENT:**

Protocol of study "*ethanol-lock* " Page 1 of 14

***EVALUATION OF ICLUSION***

***INCLUSION CRITERIA* YES—NO**

***1. Age in years ≥18.***

***2. Recent MHS with Central Vascular Catheters (CVC) inserted>48 hours.***

***3. No evidence or suspicion of CR-BSI at enrollment***

***EXCLUSION CRITERIA***

***1. Pregnancy***

***2. History of allergy or intolerance to ethanol.***

***3. Chronic Liver disease***

***.***

***If yes or no, check that apply***

***Date of Inclusion :* ____/____/________**

**Signature: _______________________________**

***A. PATIENT IDENTIFICATION DATA* RAMDOMIZATION**

**A B**

Protocol of study " *ethanol-lock* " Page 3 of 14

**1. Name : ______________________________________________________**

**2. Date of birth: __/__/____ 3. Sex (0: Male; 1: Female):**

**4. Medical record number: _________________**

**5. Date of admission hospital: __/__/____**

**6. Unit : __________________**

**7. Date of admission Unit: __/__/____**

**8. Date of discharge from Unit: __/__/____**

**9. Date of discharge hospital: ____/____/________**

**10. Reason for admission: ________________________________________________________**

**11. Type of Unit:**

**(0: Médical; 1: oncology; 2 surgical; 3: ICU; 4: others)**

**12. Admission (0: urgent; 1: programmed)**

**13. ¿Admission from another hospital? (0: NO; 1: YES)**

**Admission date: __/__/____**

Number of bed:

***B. GENERAL CLINICAL DATA***

Protocol of study " *ethanol-lock* " Page 3 of 14

**13. Summary of the clinical history:**

**:**

**14. *Disease Severity Classification* *** ( McCabe and Jackson):**

|  | **Classification** |
| --- | --- |
| **not expected to survive more than *1 year*** | **1 Rapidly fatal** |
| **not expected to survive more than *5 years*** | **2 Ultimately fatal** |
| **5 year survival not affected by underlying disease** | **3 Nonfatal** |

**15. Charlson’s comorbidity index:**

| **1 point** | Yes | No | **2 points** | Yes | No |
| --- | --- | --- | --- | --- | --- |
| Myocardial infarct |  |  | Hemiplegia |  |  |
| congestive heart failure |  |  | moderate or severe kidney disease |  |  |
| peripheral vascular disease |  |  | diabetes with end organ damage |  |  |
| cerebrovascular disease |  |  | tumor |  |  |
| dementia |  |  | leukemia |  |  |
| chronic lung disease |  |  | lymphoma |  |  |
| connective tissue disease |  |  |  |  |  |
| ulcer |  |  |  |  |  |
| chronic liver disease |  |  |  |  |  |
| diabetes |  |  |  |  |  |
| **3 points** | Yes | No | **6 points** | Yes | No |
| Moderate or severe liver disease |  |  | Malignant tumor, metastases |  |  |
|  | | | AIDS |  |  |

**16. Neutropenia (< 500 PMN/μL) (0: No; 1: Yes):**

**17. Surgery at admission (0: No; 1: Yes):**

**Date of surgery: ____/____/________ Type of surgery: ________________**

Protocol of the study " *ethanol-lock* " Page 4 of 14

**18. APACHE II score: A +B +C =**

| **A** | **+4** | **+3** | **+2** | **+1** | **0** | **+1** | **+2** | **+3** | **+4** |
| --- | --- | --- | --- | --- | --- | --- | --- | --- | --- |
| **Temperature** | >=41 | 39-40,9 |  | 38,5-38,9 | 36-38,4 | 34-35,9 | 32-33,9 | 30-31,9 | ≤29,9 |
| **Mean Arterial Pressure** | >160 | 130-159 | 110-129 |  | 70-109 |  | 50-69 |  | ≤49 |
| **Heart rate** | >180 | 140-179 | 110-139 |  | 70-109 |  | 55-69 | 40-54 | ≤39 |
| **Respiratory heart** | >50 | 35-49 |  | 25-34 | 12-24 | 10-11 | 6-9 |  |  |
| **FI02>=0.5 A-AdO2** | >500 | 350-499 | 200-349 |  | <200 |  |  |  |  |
| **FI02<0.5 PaO2** |  |  |  |  | >70 | 61-70 |  | 55-60 | <55 |
| **Arterial pH** | >7,7 | 7,6-7,69 |  | 7,5-7,59 | 7,33-7,49 |  | 7,25-7,32 | 7,15-7,24 | <7,15 |
| **Serum Sodium** | >180 | 160-179 | 155-159 | 150-154 | 130-149 |  | 120-129 | 111-119 | ≤110 |
| **Serum Potassium** | >7 | 6-6,9 |  | 5,5-5,9 | 3,5-5,4 | 3-3,4 | 2,5-2,9 |  | <2,5 |
| **Serum Creatinine** | >3,5 | 2-3,4 | 1,5-1,9 |  | 0,6-1,4 |  | <0,6 |  |  |
| **Hematocrit (%)** | >60 |  | 50-59,9 | 46-49,9 | 30-45,9 |  | 20-29,9 |  | <20 |
| **White Blood Count** | >40 |  | 20-39,9 | 15-19,9 | 3-14,9 |  | 1-2,9 |  | <1 |

| **B** | **+1** | **+2** | **+3** | **+5** | **+6** |
| --- | --- | --- | --- | --- | --- |
| **Age** | ≤ 44 years | 45-54 years | 55-64 years | 65-74 years | ≥ 75 years |

| **C** | Chronic Health Points: If the patient has a history of severe organ system insufficiency or is immunocompromised as defined below, assign points as follows:   - **5 points** for non-operative or emergency postoperative patients - **2 points** for elective postoperative patients   **Definitions:** organ insufficiency or immunocompromised state must have been evident prior to this hospital admission and conform to the following criteria:   - **Liver** – biopsy proven cirrhosis and documented portal hypertension; episodes of past upper GI bleeding attributed to portal hypertension; or prior episodes of hepatic failure/encephalopathy/coma.**Cardiovascular** – New York Heart Association Class IV.**Respiratory** – Chronic restrictive, obstructive, or vascular disease resulting in severe exercise restriction (i.e., unable to climb stairs or perform household duties; or documented chronic hypoxia, hypercapnia, secondary polycythemia, severe pulmonary hypertension (>40 mmHg), or respirator dependency.**Renal** – receiving chronic dialysis.**Immunocompromised** – the patient has received therapy that suppresses resistance to infection (e.g., immunosuppression, chemotherapy, radiation, long term or recent high dose steroids, or has a disease that is sufficiently advanced to suppress resistance to infection, e.g., leukemia, lymphoma, AIDS) |
| --- | --- |

***C. DATOS CLÍNICOS ESPECÍFICOS DEL ESTUDIO***

Protocol of the study " *ethanol-lock* " Page 5 of 14

**19. *Euro*SCORE: 20. Data of heart surgery:____________**

**.- ASA**

**.- Urgent indication (0: No; 1:Yes)**

**.- Cardiopulmonary by-pass time: ___minutes**

**.- Aortic cross-clamptime:___minutes**

**.- Surgery time:___minutes**

**.- Reintervention (0: No; 1: Yes)**

**.- Surgical incidents:**

**______________________________**

**______________________________**

**______________________________ ______________________________ ______________________________ .- Surgical antibiotics antibiotic**

**(0: No; 1: Yes)**

**
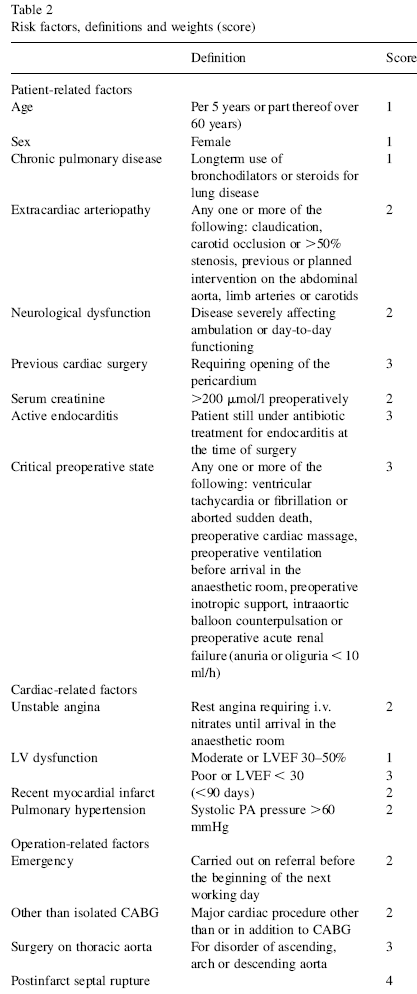
**

| **Drug** | **Doses** | **Start Date** | **Final Date** |
| --- | --- | --- | --- |
|  |  |  |  |
|  |  |  |  |
|  |  |  |  |

**0-2: Bajo riesgo; 3-5: Riesgo moderado; ≥6: Alto riesgo**

| ***Fármaco*** | ***Vía*** | ***Dosis*** | ***Fecha inicio*** | ***Fecha fin*** |
| --- | --- | --- | --- | --- |
|  |  |  |  |  |
|  |  |  |  |  |
|  |  |  |  |  |
|  |  |  |  |  |

**0-2: Low Risk**

**3-5: Medium Risk**

**≥ 6: High Risk**

***D. DATA MONITORING AND EVALUATION***

Protocol of study " *ehtanol-lock* " Page 6 of 14

**21.** **Data on Central Venous Catheter (INCLUDE ONE PAGE/CATHETER)**

Number of CVC :

*** Nº of CVCs (1,2,3):**

**.- Date of insertion: ____/____/________**

**.- Date of withdrawal: ____/____/________**

**.- Date of reception : ____/____/________**

**.- Type of Catheter**

**(0: CVC conventional; 1: Introductor of Swan-Ganz; 2: Shaldon hemodyalisis; 3: Peripherally inserted central (PIC); 4: Others:________________________)**

**.- Number of lumens:**

**.- Tunneled catheter (0: No; 1:Yes)**

**.- Parenteral Nutrition (NPT) (0: No; 1: Yes):**

**.- Entry site (0: subclavia; 1: yugular; 2: femoral; 3: others:_______________)**

**.- Material of catheter (0: polyurethane; 1: silicone; 2: others:___________)**

**.- Reason for catheter withdrawal (0: suspicion of infection; 1: end of use; 2: obstruction; 4: others:_________________________)**

*** If the patient has more catheters, you must attach a page for each catheter.**

**22. Data of E-lock surveillance**

Protocol of study " *ehtanol-lock* " Page 7 of 14

**. Patient: ____________________________________________**

**. Service of admission:­­ ____________________________________**

**. Group of randomization: A B**

**. Number of CVC:**

**TABLE OF LOCK SOLUTIONS**

| **DATE** | **Start time lock** | **Lumen lock^1^** | **Final time Lock** | **NPT(lumen lock^2^** | **Adverse events with lock*^2^** | **Lock solution^2^** | **Incidences^3^** | **Signature of responsable person** |
| --- | --- | --- | --- | --- | --- | --- | --- | --- |
| _/_/_ | __:__ |  | __:__ | **YES—NO** | **YES—NO** |  |  |  |
| _/_/_ | __:__ |  | __:__ | **YES—NO** | **YES—NO** |  |  |  |
| _/_/_ | __:__ |  | __:__ | **YES—NO** | **YES—NO** |  |  |  |
| _/_/_ | __:__ |  | __:__ | **YES—NO** | **YES—NO** |  |  |  |
| _/_/_ | __:__ |  | __:__ | **YES—NO** | **YES—NO** |  |  |  |
| _/_/_ | __:__ |  | __:__ | **YES—NO** | **YES—NO** |  |  |  |
| _/_/_ | __:__ |  | __:__ | **YES—NO** | **YES—NO** |  |  |  |
| _/_/_ | __:__ |  | __:__ | **YES—NO** | **YES—NO** |  |  |  |

Protocol of study " *ethanol-lock* " Page 8 of 14

**^1^ Type of lumen catheter: PROXIMAL, MEDIAL, MEDIAL-1, MEDIAL-2, MEDIAL-3, DISTAL.**

**^2^ In the answers YES—NO, check the correct.**

*** If adverse events after of lock, comment in the issue nº 26 of the protocol.**

**23.Clinical Data of Central Venous Central(CVC) Related Infection**

Protocol of study " *ethanol-lock* " Page 9 of 14

| **DATE OF SURVEILLANCE** | **DATA OF INFECTION OF ENTRY SITE** | **DATA OF SEPSIS** | **OTHER INFECTION** |
| --- | --- | --- | --- |
|  |  |  |  |
|  |  |  |  |
|  |  |  |  |
|  |  |  |  |
|  |  |  |  |
|  |  |  |  |
|  |  |  |  |
|  |  |  |  |
|  |  |  |  |
|  |  |  |  |
|  |  |  |  |
|  |  |  |  |
|  |  |  |  |
|  |  |  |  |
|  |  |  |  |
|  |  |  |  |
|  |  |  |  |
|  |  |  |  |
|  |  |  |  |
|  |  |  |  |
|  |  |  |  |
|  |  |  |  |

**Data of surveillance:**

Protocol of study " *ethanol-lock* " Page 10 of 14

**1. Entry site**

a. Eyithema

b. Induration

c. Pain

d. > Temperature

e. Purulent exudate

**2. Data of sepsis**

a. Temperature

b. Arterial Pressure

- Need of inotropics

c. Heart rate

d. Respiratory Rate/Pa CO_2_

f. White Blood Count/% PMNs

**3. Other infection**

**(non-related to CVC)**

a. Respiratory

b. Urinary tract infection

c. Abdominal

d. Surgical wound Infection

e. Others (specify)

**24. Episode of CR-BSI**

Protocol of study " *ethanol-lock* " Page 11 of 14

**. Clinical suspicion (0: No; 1: Yes)**

**. Date of suspicion: ____/____/________**

**. Blood cultures (0: No; 1: Yes)**

**. Date of blood culture: ____/____/________**

**. Nº of blood cultures:**

**. Type of blood cultures:**

**Diferential time Peripheral**

**. Result: Sterile Contaminated Positive**

**(microorganism: ___________________________)**

**. Catheter withdrawal (0: No; 1: Yes)**

**. Result culture CVC (check the correct):**

**Sterile Non-significant count Significant count**

**Same species than blood culture Different species than blood cultures**

**25.Antibiotic treatment in the study period**

| ***Drug*** | ***IV/ORAL*** | ***Doses*** | ***Date of start*** | ***Date of finish*** |
| --- | --- | --- | --- | --- |
|  |  |  |  |  |
|  |  |  |  |  |
|  |  |  |  |  |
|  |  |  |  |  |

**26. Adverse events attributable to E-lock (0: No; 1: Yes)**

**(Check that apply)**

Protocol of study " *ethanol-lock* " Page 12 of 14

**. Headache**

**. Nausea-vomits**

**. Asthenia**

**. Equilibrium alteration**

**. Photopsies**

**.**

**. Liver Alterations**

|  | **Day 0** | **Day +2** | **Day +4** | **Day +6** | **Day +8** | **Day**  **+10** | **Day +12** | **Day +14** | **Day +16** | **Day +18** | **Day +20** | **Day +22** | **Day +24** | **Day +26** |
| --- | --- | --- | --- | --- | --- | --- | --- | --- | --- | --- | --- | --- | --- | --- |
| **AST/ALT** |  |  |  |  |  |  |  |  |  |  |  |  |  |  |
| **GGT** |  |  |  |  |  |  |  |  |  |  |  |  |  |  |
| **Bilirrubin** |  |  |  |  |  |  |  |  |  |  |  |  |  |  |
| **Others** |  |  |  |  |  |  |  |  |  |  |  |  |  |  |

**. Neurological Alterations**

**- Seizures**

**- Delirium**

**- Others: ____________________________________________________**

**. Others adverse events (specify): ____________________________________________________________________________________________________________________________________________________________________________________________________________________________________________________________**

***E.FINAL EVOLUTION DATA***

Protocol of study "*ethanol-lock* " Page 13 of 14

**27. Others Infections (0: NO; 1: YES**)

| **INFECTIÓN** | **Type of infection*** | **MICROORGANISMS** | **DATE** |
| --- | --- | --- | --- |
| **Bacteriemia** |  |  | **__/__/____** |
| **Surgical wound Infection** |  |  | **__/__/____** |
| **Respiratory Infection**  **(no associated to mechanical ventilation)** |  |  | **__/__/____** |
| **Ventilator Associated Pneumonia (VAP)** |  |  | **__/__/____** |
| **Urinary tract infection** |  |  | **__/__/____** |
| **Clostridium difficile-associated diarrhea,**  **(CDAD)** |  |  | **__/__/____** |
| **Others (specify)** |  |  | **__/__/____** |

*** Specify episode**

**28. Final Evaluation Data**

**. 0 Live**

**. 1 Death attributable to CR-BSI**

**. 2 Death non- attributable to CR-BSI**

**. 3 Death by other cause.**

**. DDDs: daily defined doses** **:**

**. Diagnóstico de infección relacionada con el catéter:**

**0: *No infection*; 1: *Contamination*; 2: *Colonization*; 3: *Infection of entry site*; 4: CR-BSI.**

**. Episodes of CDAD (0: NO; 1: YES):**

**. Advere events attribuitable to ethanol (0: NO; 1: YES)**

**. Days with antibiotics:**

***F. MICROBIOLÓGICAL DIAGNOSIS DATA***

Protocol of study " *ethanol-lock* " Page 14 of 14

**1. MICROBIOLOGY SAMPLES**

**A. Samples of CVC**

| **Type of sample** | **Date of reception** | **Result*** | **Microorganisms** | **Date of result** |
| --- | --- | --- | --- | --- |
|  | **__/__/__** |  |  | **__/__/__** |
|  | **__/__/__** |  |  | **__/__/__** |
|  | **__/__/__** |  |  | **__/__/__** |
|  | **__/__/__** |  |  | **__/__/__** |
|  | **__/__/__** |  |  | **__/__/__** |
|  | **__/__/__** |  |  | **__/__/__** |
|  | **__/__/__** |  |  | **__/__/__** |
|  | **__/__/__** |  |  | **__/__/__** |
|  | **__/__/__** |  |  | **__/__/__** |
|  | **__/__/__** |  |  | **__/__/__** |

*** SC (significant count), NSC (non-significant count), Sterile.**

**B. Blood cultures**

| **Type** | **Date of reception** | **Result^1^** | **Interpretation** | **Microorganisms** | **Nº archivo** | **Date** |
| --- | --- | --- | --- | --- | --- | --- |
|  | **__/__/__** |  |  |  |  | **__/__/__** |
|  | **__/__/__** |  |  |  |  | **__/__/__** |
|  | **__/__/__** |  |  |  |  | **__/__/__** |
|  | **__/__/__** |  |  |  |  | **__/__/__** |
|  | **__/__/__** |  |  |  |  | **__/__/__** |
|  | **__/__/__** |  |  |  |  | **__/__/__** |
|  | **__/__/__** |  |  |  |  | **__/__/__** |
|  | **__/__/__** |  |  |  |  | **__/__/__** |
|  | **__/__/__** |  |  |  |  | **__/__/__** |
|  | **__/__/__** |  |  |  |  | **__/__/__** |

**^1^Results: POS (positive), NEG (estéril), CONT (contaminated)**
