# Supplementary material for: Ethanol Lock Therapy (E-Lock) in the Prevention of Catheter-Related Bloodstream Infections (CR-BSI) after Major Heart Surgery (MHS): A Randomized Clinical Trial
Source: PLoS One. 2014 Mar 27;9(3):e91838. doi: 10.1371/journal.pone.0091838 (PMC3967996; doi:10.1371/journal.pone.0091838)
Supplement: Protocol S2 — Spanish Translation of Protocol S1. (DOC) [file pone.0091838.s003.doc]

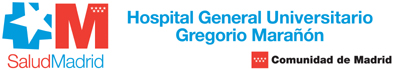


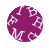


**CUADERNO DE RECOGIDA DE DATOS (CRD) DEL ESTUDIO TITULADO:**

**“*Estudio de la terapia de cierre con etanol en la prevención de la infección relacionada con catéteres venosos centrales*”**

**Servicio de Microbiología Clínica y Enfermedades Infecciosas**

**Unidad de Cuidados Post-cirugía Cardiaca**

**Hospital General Universitario Gregorio Marañón**

**Nº DE PROTOCOLO: Nº DE PACIENTE:**

Protocolo datos estudio "*Profilaxis IRC etanol-lock* " Página 1 de 14

***EVALUACIÓN DE INCLUSIÓN EN EL ESTUDIO***

***CRITERIOS DE INCLUSIÓN* SÍ—NO**

***1. Paciente adulto (≥18 años de edad).***

***2. Ser portador de un CVC durante al menos 48***

***horas antes de la evaluación de la inclusión.***

***3. El paciente (o su representante) ha otorgado su consentimiento informado.***

***CRITERIOS DE EXCLUSIÓN***

***1. Embarazo***

***2. Historia previa de alergia al etanol***

***3. Insuficiencia hepática grave***

***Para poder ser incluido en el estudio, deben cumplirse todos los criterios de inclusión y ninguno de los de exclusión.***

***Si se cumple lo anterior, continuar con los siguientes puntos del protocolo.***

***FECHA DE INCLUSIÓN:* ____/____/________**

**Hoja cumplimentada por: _______________________________**

***A. DATOS DE FILIACIÓN DEL PACIENTE Grupo aleatorización***

**A B**

Protocolo datos estudio "*Profilaxis IRC etanol-lock* " Página 3 de 14

**1. Nombre y apellidos (iniciales): ______________________________________________________**

**2. Fecha nacimiento: __/__/____ 3. Sexo (0: varón; 1: mujer):**

**4. Nº Historia Clínica: _________________**

**5. Fecha ingreso hospital: __/__/____**

**6. Servicio ingreso:__________________**

**7. Fecha de ingreso en la Unidad: __/__/____**

**8. Fecha de alta de la Unidad: __/__/____**

**9. Fecha alta hospital: ____/____/________**

**10. Motivo de ingreso: ________________________________________________________**

**11. Tipo servicio de ingreso:**

**(0: Médico; 1: oncológico; 2 quirúrgico; 3: cuidados intensivos adultos; 4: pediatría no UCI; 5: neonatología no UCI; 6: cuidados intensivos pediatría o neonatología)**

**12. Tipo de ingreso (0: urgente; 1: programado)**

**13. ¿Procede de otro hospital? (0: NO; 1: SÍ)**

**Fecha ingreso: __/__/____**

Nº de cama:

***B. DATOS CLÍNICOS GENERALES (antecedentes y datos del episodio actual)***

Protocolo datos estudio " *Profilaxis IRC etanol-lock* " Página 3 de 14

**13. Breve resumen de la historia clínica:**

**14. Enfermedad de base*** (clasificación de McCabe y Jackson):**

| **Enfermedad de base** | **Ejemplo** | **Clasificación** |
| --- | --- | --- |
| Fulminante. La muerte se espera en los próximos 2 meses | Leucemia aguda en fase blástica, enfermedad rápidamente progresiva | **1** Rápidamente fatal |
| Irreversible con muerte esperada en los próximos 4 años a pesar de un tratamiento óptimo | Carcinoma metastático, linfoma maligno, fallo renal crónico sin trasplante | **2** Últimamente fatal |
| Reversible, crónica o aguda. La muerte no se espera en los 4 años siguientes | Diabetes, enfermedades obstétricas, gastrointestinales o génito-urinarias | **3** No fatal |

15. Índice ponderado de co-morbilidad (Charlson):

| **Asigne 1 punto por cada uno** | Sí | No | **Asigne 2 puntos por cada uno** | Sí | No |
| --- | --- | --- | --- | --- | --- |
| Infarto de miocardio |  |  | Hemiplejia |  |  |
| Insuficiencia cardíaca congestiva |  |  | Nefropatía moderada/ grave |  |  |
| Enfermedad vascular periférica |  |  | Diabetes con repercusión orgánica |  |  |
| Enfermedad cerebrovascular |  |  | Cualquier tumor |  |  |
| Demencia |  |  | Leucemia |  |  |
| Enfermedad pulmonar crónica |  |  | Linfoma |  |  |
| Colagenosis |  |  |  |  |  |
| Enfermedad ulcerosa |  |  |  |  |  |
| Hepatopatía leve |  |  |  |  |  |
| Diabetes |  |  |  |  |  |
| **Asigne 3 puntos por cada uno** | Sí | No | **Asigne 6 puntos por cada uno** | Sí | No |
| Hepatopatía moderada/ grave |  |  | Tumor metastásico |  |  |
|  | | | SIDA |  |  |

**Puntuación total de co-morbilidad:**

**16. Neutropenia previa (< 500 PMN/μL) (0: No; 1: Sí):**

**17. Cirugía en el ingreso actual (0: No; 1: Sí):**

**Fecha cirugía: ____/____/________ Tipo cirugía: _____________________**

Protocolo datos estudio " *Profilaxis IRC etanol-lock* " Página 4 de 14

**18. APACHE II score: A +B +C =**

| **A** | **+4** | **+3** | **+2** | **+1** | **0** | **+1** | **+2** | **+3** | **+4** |
| --- | --- | --- | --- | --- | --- | --- | --- | --- | --- |
| **Temperatura** | >=41 | 39-40,9 |  | 38,5-38,9 | 36-38,4 | 34-35,9 | 32-33,9 | 30-31,9 | ≤29,9 |
| **Presión arterial media** | >160 | 130-159 | 110-129 |  | 70-109 |  | 50-69 |  | ≤49 |
| **Frecuencia cardiaca** | >180 | 140-179 | 110-139 |  | 70-109 |  | 55-69 | 40-54 | ≤39 |
| **Frecuencia respiratoria** | >50 | 35-49 |  | 25-34 | 12-24 | 10-11 | 6-9 |  |  |
| **FI02>=0.5 A-AdO2** | >500 | 350-499 | 200-349 |  | <200 |  |  |  |  |
| **FI02<0.5 PaO2** |  |  |  |  | >70 | 61-70 |  | 55-60 | <55 |
| **pH sanguíneo** | >7,7 | 7,6-7,69 |  | 7,5-7,59 | 7,33-7,49 |  | 7,25-7,32 | 7,15-7,24 | <7,15 |
| **Na+** | >180 | 160-179 | 155-159 | 150-154 | 130-149 |  | 120-129 | 111-119 | ≤110 |
| **K+** | >7 | 6-6,9 |  | 5,5-5,9 | 3,5-5,4 | 3-3,4 | 2,5-2,9 |  | <2,5 |
| **Creatininemia *** | >3,5 | 2-3,4 | 1,5-1,9 |  | 0,6-1,4 |  | <0,6 |  |  |
| **Hematocrito** | >60 |  | 50-59,9 | 46-49,9 | 30-45,9 |  | 20-29,9 |  | <20 |
| **Leucocitosis** | >40 |  | 20-39,9 | 15-19,9 | 3-14,9 |  | 1-2,9 |  | <1 |

*** Puntuación doble en el caso de Insuficiencia Renal Aguda**

| **B** | **+1** | **+2** | **+3** | **+5** | **+6** |
| --- | --- | --- | --- | --- | --- |
| **Edad** | ≤ 44 años | 45-54 años | 55-64 años | 65-74 años | ≥ 75 años |

| **C** | Si existe **concomitantemente** Fallo Multiorgánico o Inmunocompromiso:  .Paciente no quirúrgico o post-operatorio de cirugía urgente: **5** puntos  .Post-operatorio de cirugía electiva**: 2** puntos  . La **insuficiencia orgánica** *debe haberse evidenciado antes del ingreso actual e incluye: HIGADO (cirrosis probada histológicamente o con episodios de descompensación), CORAZÓN (NYHA clase IV), PULMÓN (I. Crónica restrictiva, obstructiva o vascular resultado en restricción grave al esfuerzo o hipoxia crónica documentada, hipercapnia, policitemia, hipertensión pulmonar >40 mmHg o dependencia del respirador), RIÑON (diálisis crónica).*  . **INMUNOCOMPROMISO**. *Paciente en el cual existe un riesgo aumentado de adquirir infección como consecuencia de presentar una anormalidad (adquirida o congénita) en el sistema inmune: Si el paciente recibe ≥ 5mg/d de esteroides, radioterapia, si el paciente es VIH+, padece leucemia o linfoma, es receptor de transplante de órgano sólido o MO, si ha recibido tto con drogas inmunosupresoras al menos 30 días previos.* |
| --- | --- |

***C. DATOS CLÍNICOS ESPECÍFICOS DEL ESTUDIO***

Protocolo datos estudio " *Profilaxis IRC etanol-lock* " Página 5 de 14

**19. *Euro*SCORE: 20. Datos cirugía cardiaca:**

**.- Índice ASA***

**.- Indicación (0: electiva; 1: urgente)**

**.- Tiempo de CEC:___minutos**

**.- Tiempo clampado aórtico:___minutos**

**.- Tiempo total intervención:___minutos**

**.- Reintervención (0: No; 1: Sí)**

**.- Incidentes quirúrgicos:**

**______________________________**

**______________________________**

**______________________________ ______________________________ ______________________________ .- Profilaxis quirúrgica antibiótica**

**(0: No; 1: Sí)**

**
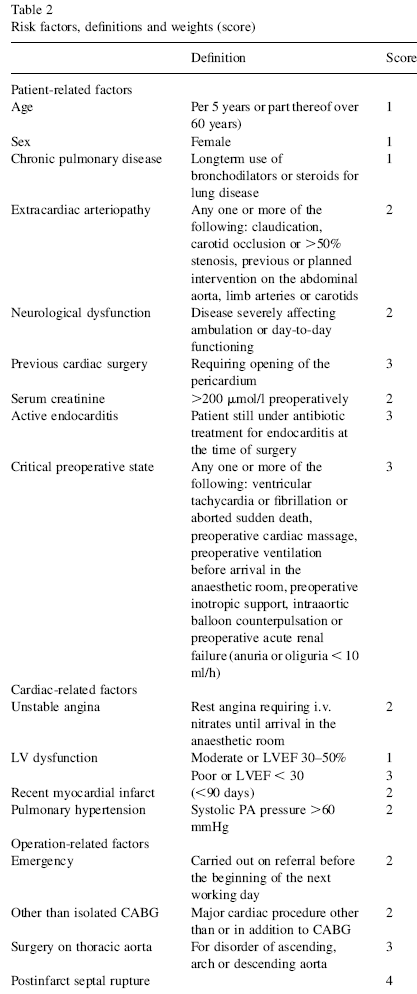
**

| **Fármaco** | **Dosis/Vía** | **Fecha inicio** | **Fecha fin** |
| --- | --- | --- | --- |
|  |  |  |  |
|  |  |  |  |
|  |  |  |  |

**0-2: Bajo riesgo; 3-5: Riesgo moderado; ≥6: Alto riesgo**

| ***Fármaco*** | ***Vía*** | ***Dosis*** | ***Fecha inicio*** | ***Fecha fin*** |
| --- | --- | --- | --- | --- |
|  |  |  |  |  |
|  |  |  |  |  |
|  |  |  |  |  |
|  |  |  |  |  |

***ÍNDICE ASA**

**1:** Sano, **2**: Moderado, **3**: Grave no vital, **4**: Grave riesgo vital, **5:** Riesgo de muerte inminente

**0-2: Bajo Riesgo**

**3-5: Riesgo Moderado**

**≥ 6: Alto Riesgo**

***D. DATOS DE SEGUIMIENTO Y EVALUACIÓN***

Protocolo datos estudio " *Profilaxis IRC etanol-lock* " Página 6 de 14

**21.** **Datos del Catéter Venoso Central (INCLUIR UNA HOJA POR CATETER)**

Número del CVC en este paciente:

*** Nº de CVCs del paciente (1,2,3):**

**.- Fecha de inserción: ____/____/________**

**.- Fecha de retirada: ____/____/________**

**.- Fecha recepción laboratorio: ____/____/________**

**.- Tipo de Catéter**

**(0: CVC convencional; 1: Swan-Ganz; 2: Shaldon hemodiálisis; 3: CVC insertado periféricamente; 4: Otros:________________________)**

**.- Número de luces:**

**.- CVC tunelizado (0: No; 1: Sí)**

**.- Nutrición parenteral (NPT) (0: No; 1: Sí):**

**.- Lugar inserción (0: subclavia; 1: yugular; 2: femoral; 3: otros:_______________)**

**.- Material del catéter (0: poliuretano; 1: silicona; 2: otros:___________)**

**.- Motivo de retirada (0: sospecha de infección; 1: fin de uso; 2: obstrucción/mal función; 4: otros:_________________________)**

*** Si el paciente tiene más de un CVC puesto en el momento de la evaluación, se considerará el hecho y se rellenará otra hoja como a presente con los datos del resto de CVCs que tenga el paciente.**

**22. Datos de seguimiento de aplicación de la solución de sellado**

Protocolo datos estudio " *Profilaxis IRC etanol-lock* " Página 7 de 14

**. Paciente: ____________________________________________**

**. Unidad de ingreso:­­ ____________________________________**

**. Grupo de aleatorización (señalar el que proceda): A B**

**. Número del CVC (en caso de que el paciente tenga más de**

**un CVC puesto):**

TABLA SEGUIMIENTO ADMINISTRACIÓN SOLUCIÓN DE SELLADO

| **Fecha** | **Hora inicio sellado** | **Luz sellada^1^** | **Hora fin sellado** | **NPT por luz sellada^2^** | **Efectos 2^arios^ con infusión*^2^** | **Producto**  **Sellado^2^** | **Comentarios/incidencias^3^** | **Firma responsable administración** |
| --- | --- | --- | --- | --- | --- | --- | --- | --- |
| _/_/_ | __:__ |  | __:__ | **SI—NO** | **SI—NO** |  |  |  |
| _/_/_ | __:__ |  | __:__ | **SI—NO** | **SI—NO** |  |  |  |
| _/_/_ | __:__ |  | __:__ | **SI—NO** | **SI—NO** |  |  |  |
| _/_/_ | __:__ |  | __:__ | **SI—NO** | **SI—NO** |  |  |  |
| _/_/_ | __:__ |  | __:__ | **SI—NO** | **SI—NO** |  |  |  |
| _/_/_ | __:__ |  | __:__ | **SI—NO** | **SI—NO** |  |  |  |
| _/_/_ | __:__ |  | __:__ | **SI—NO** | **SI—NO** |  |  |  |
| _/_/_ | __:__ |  | __:__ | **SI—NO** | **SI—NO** |  |  |  |

Protocolo datos estudio " *Profilaxis IRC etanol-lock* " Página 8 de 14

**^1^ Elegir, según el número de luces del CVC, entre: PROXIMAL, MEDIAL, MEDIAL-1, MEDIAL-2, MEDIAL-3, DISTAL.**

**^2^ En las respuestas SI—NO, rodear con un círculo la correcta. Rodear también la opción de tratamiento asignado que corresponda.**

**^3^ Registrar en esta columna si ha sido necesario disminuir la concentración de administración de etanol del 70% al 25%.**

*** Si se producen efectos secundarios tras infundir la solución de sellado, especificarlos en el apartado nº 26 del protocolo.**

**Dejar esta hoja de seguimiento en la historia del paciente, para poder ser cumplimentada por la enfermera encargada de la administración de la solución de sellado. Una vez retirado el CVC, adjuntar al protocolo original.**

**23. Datos clínicos de Infección Relacionada con CVC**

Protocolo datos estudio " *Profilaxis IRC etanol-lock* " Página 9 de 14

| **DÍA DE SEGUIMIENTO** | **DATOS INFECCIÓN SITIO INSERCIÓN** | **DATOS DE SEPSIS** | **OTRO FOCO INFECCIOSO PROBABLE** |
| --- | --- | --- | --- |
|  |  |  |  |
|  |  |  |  |
|  |  |  |  |
|  |  |  |  |
|  |  |  |  |
|  |  |  |  |
|  |  |  |  |
|  |  |  |  |
|  |  |  |  |
|  |  |  |  |
|  |  |  |  |
|  |  |  |  |
|  |  |  |  |
|  |  |  |  |
|  |  |  |  |
|  |  |  |  |
|  |  |  |  |
|  |  |  |  |
|  |  |  |  |
|  |  |  |  |
|  |  |  |  |
|  |  |  |  |

*** Anotar el día y marcar con una cruz el o los datos que se detecten (señalados en la tabla).**

**Los datos que deben vigilarse son los siguientes:**

Protocolo datos estudio " *Profilaxis IRC etanol-lock* " Página 10 de 14

**1. Aspecto del sitio de inserción**

a. Eritema

b. Induración

c. Dolor

d. Aumento Tª

e. Exudado purulento

**2. Datos de sepsis**

a. Tª corporal

b. Presión arterial

- Necesidad de inotropos

c. Frecuencia cardiaca

d. Frecuencia respiratoria/Pa CO_2_

f. Leucocitos/% PMNs

**3. Otro foco infeccioso probable**

**(al margen del CVC)**

a. Respiratorio

b. Urinario

c. Abdominal

d. Herida quirúrgica

e. Otros (especificar)

**24. Episodio de BRC**

Protocolo datos estudio " *Profilaxis IRC etanol-lock* " Página 11 de 14

**. Sospecha clínica (0: No; 1: Sí)**

**. Fecha de la sospecha: ____/____/________**

**. Hemocultivos (0: No; 1: Sí)**

**. Fecha de toma: ____/____/________**

**. Nº de hemocultivos:**

**. Tipo de hemocultivos:**

**Diferencial tiempo Vía periférica**

**. Resultado: Estéril Contaminado Positivo**

**(microorganismos: ___________________________)**

**. Retirada del CVC (0: No; 1: Sí)**

**. Resultado cultivo CVC (marcar lo que proceda):**

**Estéril Recuento no significativo Recuento significativo**

**Misma especie que HC Distinta especie que HC**

**25. Tratamiento antibiótico administrado en el período de estudio**

| ***Fármaco*** | ***Vía*** | ***Dosis*** | ***Fecha inicio*** | ***Fecha fin*** |
| --- | --- | --- | --- | --- |
|  |  |  |  |  |
|  |  |  |  |  |
|  |  |  |  |  |
|  |  |  |  |  |

**26. Efectos adversos atribuibles a la solución de etanol (0: No; 1: Sí)**

**(Marque los que proceda)**

Protocolo datos estudio " *Profilaxis IRC etanol-lock* " Página 12 de 14

**. Cefalea**

**. Náuseas-vómitos**

**. Astenia**

**. Alteración equilibrio**

**. Acúfenos**

**. Fotofobia**

. Alteraciones hepáticas

|  | **Día 0** | **Día +2** | **Día +4** | **Día +6** | **Día +8** | **Día +10** | **Día +12** | **Día +14** | **Día +16** | **Día +18** | **Día +20** | **Día +22** | **Día +24** | **Día +26** |
| --- | --- | --- | --- | --- | --- | --- | --- | --- | --- | --- | --- | --- | --- | --- |
| **GOT/GPT** |  |  |  |  |  |  |  |  |  |  |  |  |  |  |
| **GGT** |  |  |  |  |  |  |  |  |  |  |  |  |  |  |
| **Bilirrubina** |  |  |  |  |  |  |  |  |  |  |  |  |  |  |
| **Otros** |  |  |  |  |  |  |  |  |  |  |  |  |  |  |

**. Alteraciones neurológicas**

**- Crisis convulsivas**

**- Delirio**

**- Otras: ____________________________________________________**

**. Otros posibles efectos adversos (especificar): ____________________________________________________________________________________________________________________________________________________________________________________________________________________________________________________________**

***E. DATOS DE EVOLUCIÓN FINAL***

Protocolo datos estudio " *Profilaxis IRC etanol-lock* " Página 13 de 14

**27. Otras infecciones durante el seguimiento** (0: NO; 1: SÍ)

| **INFECCIÓN** | **Tipo infección*** | **MICROORGANISMOS** | **FECHA** |
| --- | --- | --- | --- |
| **Bacteriemia significativa** |  |  | **__/__/____** |
| **Infección Herida Quirúrgica** |  |  | **__/__/____** |
| **Infección respiratoria**  **(no asociada a ventilación**  **mecánica)** |  |  | **__/__/____** |
| **Neumonía Asociada a Ventilación Mecánica (NAV)** |  |  | **__/__/____** |
| **Infección Urinaria Nosocomial** |  |  | **__/__/____** |
| **Diarrea Asociada a *Clostridium difficile*** **(DACD)** |  |  | **__/__/____** |
| **Otras (especificar)** |  |  | **__/__/____** |

*** En la columna “Tipo de infección”, detallar el episodio infeccioso, si procede (ej. Osteomielitis cadera, Neumonía-traqueobronquitis, etc.).**

**28. Datos de evaluación final**

**. 0 Vivo al alta de la Unidad de Cuidados Intensivos**

**. 1 Muerte atribuible a la Bacteriemia Relacionada con**

**Catéter (BRC)**

**. 2 Muerte con BRC pero atribuible a otra causa**

**. 3 Muerte por otra causa (sin BRC).**

**2: Muerte con BRC pero atribuible a otra causa: Cuando fallece el paciente por otras causas claras mientras estaba en tratamiento adecuado para BRC.**

**. Nº de DDDs de antibióticos hasta el alta:**

**. Diagnóstico de infección relacionada con el catéter:**

**0: *No infección*; 1: *Contaminación*; 2: *Colonización*; 3: *Infección del punto de entrada*; 4: *Bacteriemia Relacionada con CVC*.**

**. Episodios de DACD (0: NO; 1: SÍ):**

**. Efectos adversos atribuibles al etanol (0: NO; 1: SÍ)**

**. Días con antibióticos hasta el alta:**

***F. DATOS DEL DIAGNÓSTICO MICROBIOLÓGICO***

Protocolo datos estudio " *Profilaxis IRC etanol-lock* " Página 14 de 14

**1. MUESTRAS MICROBIOLOGÍA**

**A. Muestras del CVC**

| **Tipo muestra** | **Fecha recepción** | **Resultado*** | **Microorganismos** | **Fecha archivo** |
| --- | --- | --- | --- | --- |
|  | **__/__/__** |  |  | **__/__/__** |
|  | **__/__/__** |  |  | **__/__/__** |
|  | **__/__/__** |  |  | **__/__/__** |
|  | **__/__/__** |  |  | **__/__/__** |
|  | **__/__/__** |  |  | **__/__/__** |
|  | **__/__/__** |  |  | **__/__/__** |
|  | **__/__/__** |  |  | **__/__/__** |
|  | **__/__/__** |  |  | **__/__/__** |
|  | **__/__/__** |  |  | **__/__/__** |
|  | **__/__/__** |  |  | **__/__/__** |

*** Opciones de resultado: RS (recuento significativo), RNS (recuento no significativo), Estéril.**

B. Hemocultivos

| **Tipo** | **Nº registro** | **Fecha recepción** | **Resultado^1^** | **Interpretación^2^** | **Microorganismos** | **Fecha archivo** |
| --- | --- | --- | --- | --- | --- | --- |
|  |  | **__/__/__** |  |  |  | **__/__/__** |
|  |  | **__/__/__** |  |  |  | **__/__/__** |
|  |  | **__/__/__** |  |  |  | **__/__/__** |
|  |  | **__/__/__** |  |  |  | **__/__/__** |
|  |  | **__/__/__** |  |  |  | **__/__/__** |
|  |  | **__/__/__** |  |  |  | **__/__/__** |
|  |  | **__/__/__** |  |  |  | **__/__/__** |
|  |  | **__/__/__** |  |  |  | **__/__/__** |
|  |  | **__/__/__** |  |  |  | **__/__/__** |
|  |  | **__/__/__** |  |  |  | **__/__/__** |

**^1^Opciones de resultado: POS (positivo), NEG (estéril), CONT (contaminado)**

**^2^ En función del número de hemocultivos en cada extracción (set de HC) y el tipo (periféricos/lisis-centrifugación-diferencial de tiempo), se realizará la interpretación adecuada a las recomendaciones vigentes por parte del laboratorio.**
